# The OMM^12^ mice: a decade of dissecting host-microbiome interactions

**DOI:** 10.1080/19490976.2026.2725332

**Published:** 2026-09-22

**Authors:** Devon E. Conti, Laurent Debarbieux, Caroline Henrot

**Affiliations:** a Institut Pasteur, Université Paris Cité, UMR-CNRS 6047, Department of Microbiology, Bacteriophage Bacterium Host, Paris, France; b Sorbonne Université, Collège Doctoral, Paris, France

**Keywords:** Murine model, synthetic community, microbial functions, metabolism, bacteriophage

## Abstract

Humans and their associated microbes form a multifaceted ecosystem. Gaining mechanistic insight into this system necessitates reductionist models integrating ecological and immunological dimensions. Over the past decade, the gnotobiotic Oligo-Mouse-Microbiota (OMM^12^) mouse line has exemplified this approach, pairing a standardized consortium of 12 bacterial strains representative of the five major phyla of the mouse microbiota, with the most widely used host background, C57bl/6 mice, in microbiome research. Across numerous studies, this reproducible and experimentally tractable model has enabled the identification of molecular mechanisms underlying colonization resistance, pathogen virulence, microbiota-dependent metabolic and immune pathways, as well as bacterial and bacteriophage dynamics. The modular ability/nature of the OMM^12^ model may now be further leveraged and refined in different host and microbial dimensions to address remaining gaps in translational microbiome research, positioning it as a reference framework for mechanistic investigations.

## Introduction

Human health is intimately linked to microbes, which include bacteria, viruses (primarily bacteriophages, or phages), fungi, archaea and protozoa. This microbial community shapes many processes including nutrient metabolism, epithelial barrier function and immunity, to cite a few.^1^ ^,^ ^2^ Over the past two decades, research in this field was propelled by studies on intestinal microbiota that have progressively moved from characterizations of microbial composition to mechanistic investigations establishing a causal link between the microbiota and host physiology. A landmark example of this transition is provided by microbiota transplantation experiments, in which the transfer of microbiota from obese donors to germ-free (GF) mice increased adiposity, demonstrating that gut microbial communities can directly influence host metabolic outcomes.^3^ ^,^ ^4^

Investigations into how the intestinal microbiota shapes host physiology and intra-community interactions has predominantly relied on murine models. However, the choice of a mouse model is critical since differences in resident microbiota composition and baseline immune status can markedly influence experimental outcomes. Accordingly, model selection must be carefully aligned with the specific research question.^5–8^ To enhance reproducibility and limit confounding variables, several standardized microbiological conditions have been established. Specific pathogen-free (SPF) mice, which exclude major pathogens, offer improved reproducibility but still harbor a diverse, facility-dependant resident microbiota.^9^ ^,^ ^10^ Manipulating this microbiota often requires antibiotic treatment, for which diverse regimens exist.^7^ In contrast, germ-free (GF) mice, maintained in the complete absence of microorganisms, provide a highly simplistic model for investigating the effects of individual members or defined consortia.^11^ However, their lack of immune and physiological development limits their translational relevance, restricting their use primarily to mechanistic studies rather than investigations of community-level microbiota functions.^12^ ^,^ ^13^

To bridge this gap, defined microbial consortia, commonly referred to as synthetic communities (SynComs), have emerged as powerful tools in microbiome research. These ready-to-use assemblies, ranging from single strains to diverse collections of cultured intestinal isolates, offer standardized and precisely defined microbial compositions, enabling researchers to balance physiological relevance while maintaining experimental tractability.^14^ ^,^ ^15^ When introduced into GF mice, SynComs represent an intermediate level of microbiota complexity between GF and SPF conditions, supporting immune and physiological development while allowing tightly controlled mechanistic studies of host-microbiota interactions.^16^ ^,^ ^17^ Among the available SynComs, two have been particularly influential: the Altered Schaedler Flora (ASF) and the Oligo-Mouse-Microbiota (OMM^12^).

The ASF, established in the 1970s, consists of a stable consortium of eight murine-derived bacterial strains spanning three phyla: *Bacillota* (formerly *Firmicutes*), *Bacteroidota* (*Bacteroidetes*), and *Deferribacterota* (*Deferribacteres*). This pioneering model enabled foundational studies of host–microbe interactions, but its relatively limited microbial diversity and lack of key metabolic functions restrain its ecological relevance.^18^ ^,^ ^19^ The need for a more functionally diverse, yet still standardized and tractable community motivated the development of the OMM^12^ model, which comprises 12 murine gut isolates representing five dominant phyla of the mouse intestine—*Bacillota*, *Bacteroidota*, *Actinobacteriota* (*Actinobacteria*), *Pseudomonadota* (*Proteobacteria*), and *Verrucomicrobiota*. Over the past decade, the OMM^12^ model has been adopted in over 60 studies covering areas such as microbiota–microbiota interactions, host metabolism, and immune system regulation.^16^ ^,^ ^18^ ^,^ ^20^ This review summarizes the characterization of the OMM^12^ model and how it advanced our understanding of host-microbiota interactions.

## I - The three partners interacting in the gut of OMM^**12**^ mice

### The bacteria

#### *Composition and functional features of the 12 bacteria of the OMM* ^*12*^ *community*

**Figure 1.**
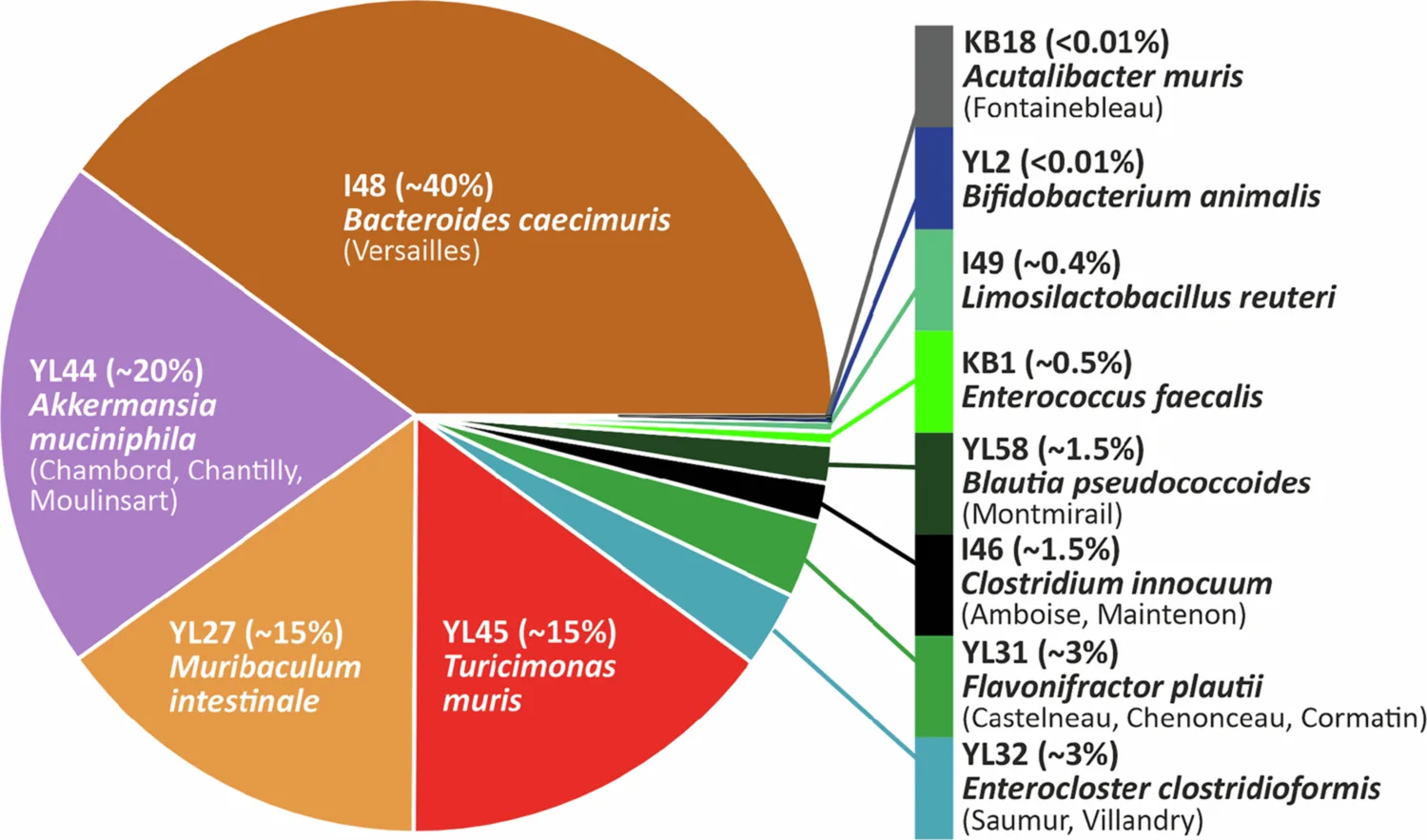
Pie chart showing the relative abundance of the 12 bacterial strains in the fecal pellets of OMM^12^ mice. Strains are arranged counterclockwise in decreasing order of abundance: *Bacteroides caecimuris* I48 (40%), *Akkermansia muciniphila* YL44 (20%), *Muribaculum intestinale* YL27 and *Turicimonas muris* YL45 (15% each), *Enterocloster clostridioformis* YL32 and *Flavonifractor plautii* YL31 (3% each), *Clostridium innocuum* I46 and *Blautia pseudococcoides* YL58 (1.5% each), *Enterococcus faecalis* KB1 (0.5%), *Limosilactobacillus reuteri* I49 (0.4%), and *Acutalibacter muris* KB18 and *Bifidobacterium animalis* YL2 (<0.01% each).Percentage were estimated from absolute abundance values determined by qPCR in previous studies.^16^ ^,^ ^25^ Color cosing is identical to that in the original figures. The 13 inducible prophages are indicated in brackets next to the corresponding strains.

The OMM^12^ model, initially referred to as the stable Defined Moderately Diverse Microbiota (sDMDMm_2_), was developed by Bärbel Stecher and colleagues by complementing GF C57BL/6 mice with 12 mouse-derived bacteria that span five of the most prevalent and abundant phyla of the murine gut and chosen for their ability to grow in laboratory conditions: *Enterococcus faecalis* KB1, *Limosilactobacillus reuteri* I49, *Bifidobacterium animalis* YL2, *Clostridium innocuum* I46, *Blautia coccoides* YL58, *Enterocloster clostridioformis* YL32, *Flavonifractor plautii* YL31, *Acutalibacter muris* KB18, *Bacteroides caecimuris* I48, *Muribaculum intestinale* YL27, *Akkermansia muciniphila* YL44 and *Turicimonas muris* YL45.^18^ ^,^ ^20^ ^,^ ^21^ The twelve bacterial isolates belong to the mouse intestinal Bacterial Collection (miBC) and stock cultures are publicly available through the German Collection of Microorganisms and Cell Cultures (DSMZ).^22^ ^,^ ^23^ Through proximity ligation sequencing (Hi-C), the 12 genomes were updated to high-quality closed assemblies.^24^ The stability of the OMM^12^ microbiota composition was then assessed at both the individual level and across generations, while the reproducibility of gut colonization was demonstrated in five facilities (Figure 1).^16^ ^,^ ^18^ The colonization procedure is standardized with oral and rectal administrations of the OMM^12^ consortium performed twice one day apart from a thawed cryostock provided by the DSMZ, while minimizing oxygen exposure.^16^

**Figure 2.**
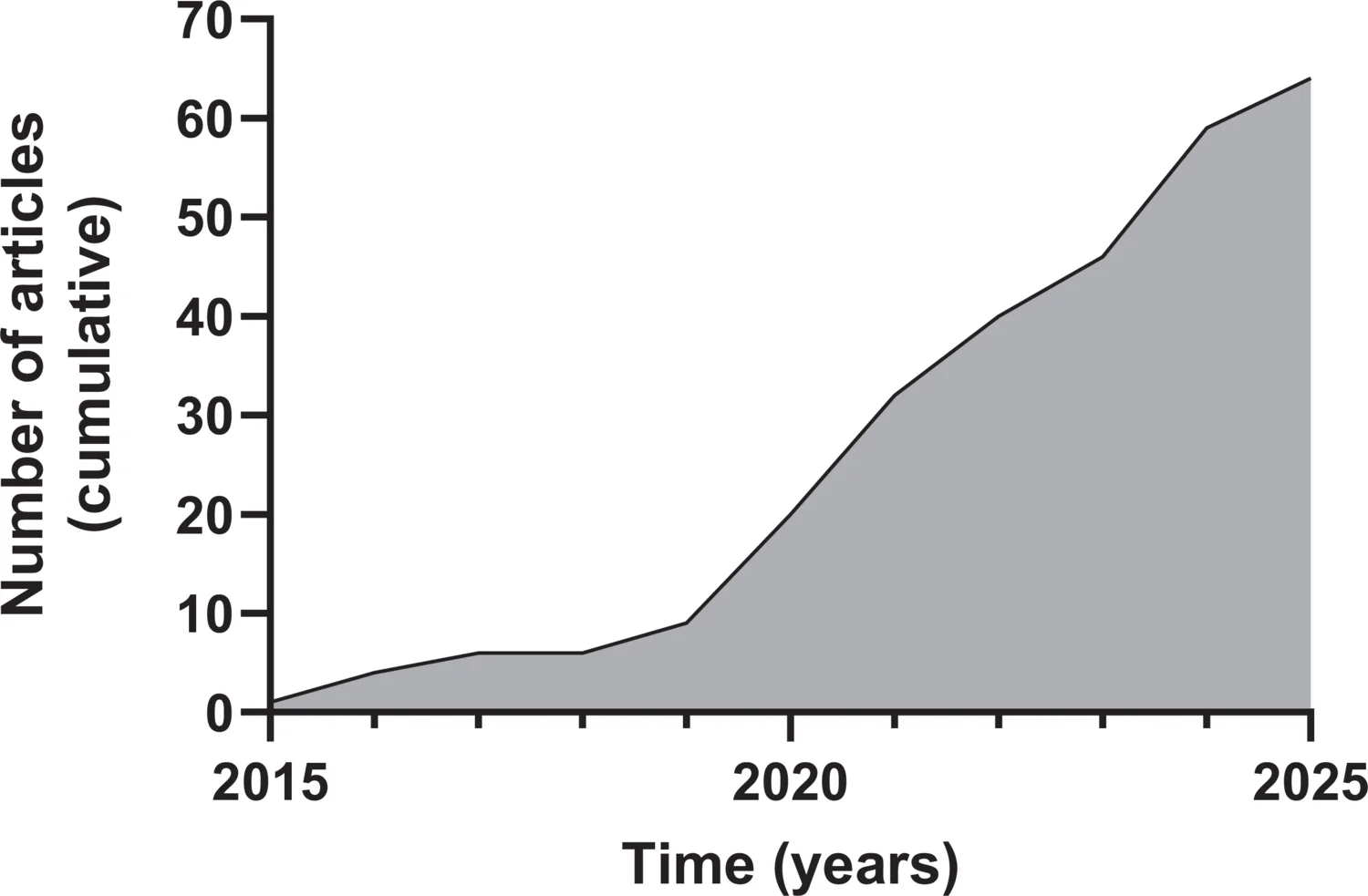
Line graph showing the cumulative number of studies using OMM^12^ mice between 2015 and 2025 (from the curated list of the 64 articles analyzed in this review). The cumulative count increased by roughly two articles per year between 2015 and 2019, and by roughly ten per year between 2020 and 2025, reflecting continuous growth with a marked acceleration in recent years.

The high reproducibility and ease of implementation of the OMM^12^ model have driven its widespread adoption in microbiome research over the past decade (Figure 2). Its reduced complexity enables precise tracking of individual community members, including spatial and temporal dynamics such as their distribution along the gastrointestinal tract, compositional development from early life to adulthood, and long-term evolutionary genomic adaptation at unprecedented resolution.^18,26–30^ Furthermore, this streamlined system facilitates mechanistic studies of microbial interactions through multi-omics approaches, allowing *in vivo* profiling of gene expression and metabolite production to dissect host-microbe relationships.^31–36^

#### Metabolic functions and cross-feedings of the 12 bacteria

Bacteria of the OMM^12^ consortium have been extensively characterized both individually and in co-cultures, enabling the identification of their metabolic profiles and interspecies interactions. Notably the production and consumption of key metabolites, including carbohydrates, bile salts, and short-chain fatty acids (SCFAs) have been mapped for each OMM^12^ member in both *in vitro* and *in vivo* settings, providing detailed insights into cross-feeding networks and community maintenance mechanisms.^37–41^

Strains of the OMM^12^ consortium exhibit paired microbial interactions spanning neutral, positive and competitive relationships. This was demonstrated through *in vitro* co-culture experiments in which the OMM^12^ community was cultivated with the omission of a single member (OMM^11^), revealing key interactions and driver strains under specific conditions. For instance, *B. caecimuris* I48 displays strong inhibitory effects against *M. intestinale* YL27 in media containing inulin or xylan as carbon source; *Enterococcus faecalis* KB1 acts as a dominant driver of OMM^12^ community composition by directly inhibiting the growth of nine consortium members in glucose-rich co-cultures.^28^ ^,^ ^41^ These interactions highlight diverse underlying mechanisms: medium acidification resulting from polysaccharide conversion by *B. caecimuris* I48 sustains the inhibition of *M. intestinale* YL27. The bacteriocin enterocin L50, produced by *Enterococcus faecalis* KB1, mediates competitive interactions with certain community members, such as *B. animalis* YL2, but only under specific medium conditions, underscoring the context-dependent nature of these interactions.^28^ Collectively, *in vitro* competitive interactions between the OMM^12^ members involve the production of anti-bacterial compounds or metabolic by-products and the direct competition for metabolites. However, the context dependence of these interactions, strongly influenced by medium composition, must be considered alongside diet-induced shifts observed *in vivo*.^30^ ^,^ ^40^ This highlights that despite the relatively low complexity of the resident bacterial community, extrapolations of mechanisms sustaining dynamics along the gastrointestinal tract from *in vitro* findings remain challenging.

**Figure 3.**
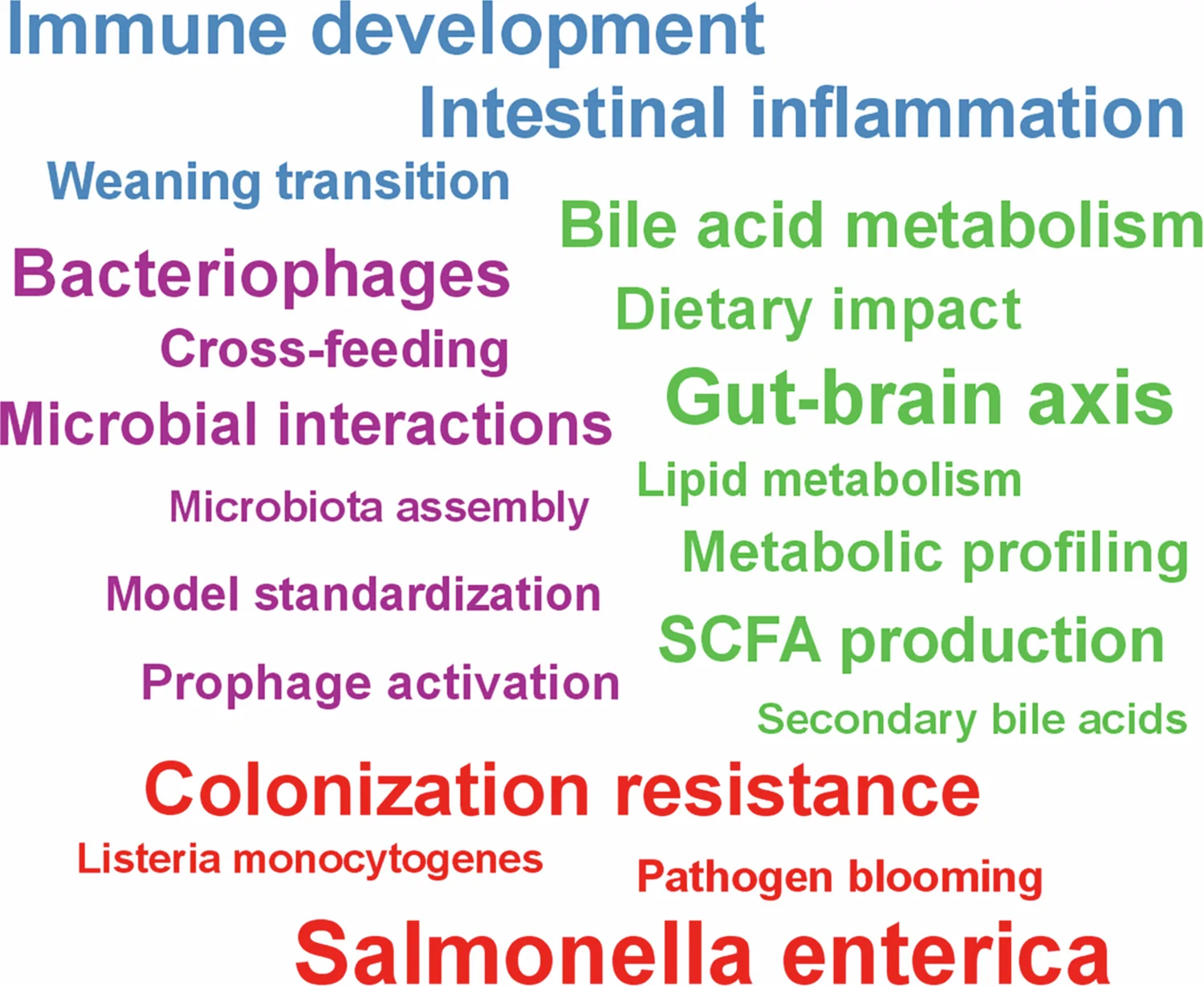
Word cloud displaying the 20 terms most frequently associated with studies with OMM^12^ mice. Gemini 3 was used to analyze the abstracts of 64 studies using OMM12 mice and identify the 20 most frequent terms. Font size is proportional to term frequency, and terms are grouped by color into four thematic clusters: blue, Host Immunity (immune development, intestinal inflammation, weaning transition); purple, Ecology/Community Structure (bacteriophages, cross-feeding, microbial interactions, microbiota assembly, model standardization, prophage activation); green, Metabolism/Physiology (bile acid metabolism, dietary impact, gut–brain axis, lipid metabolism, metabolic profiling, SCFA production, secondary bile acids); red, Infection/Resistance (colonization resistance, pathogen blooming, *Listeria monocytogenes*, *Salmonella enterica*).

Metabolic characterizations of the OMM^12^ consortium have paved the way for investigating how additional strains or dietary factors influence community metabolic states and compositional stability. In particular, metabolomic profiling of the community has enabled the targeted complementation of metabolic functions absent in the OMM^12^ model. This has facilitated the expansion of the consortium with strains performing unmet metabolic pathways, such as secondary bile acids producers (*Extibacter muris* and *Clostridium scindens*), cellulose degraders (*Alistipes finegoldii*) and taurine-respiring strains (*Taurinivorans muris*).^25^ ^,^ ^38^ ^,^ ^40^ ^,^ ^42^ ^,^ ^43^ The impact of such complementation on host physiology and overall microbiota function remains an active area of research, with further insights discussed in **Section II**.

### The prophages

The mammalian gut microbiota harbors a substantial load of viruses, with estimated abundances of 10^9^-10^10^ virus-like particles (VLPs) per gram of stools compared to 10^11^ bacterial cells.^44^ ^,^ ^45^ Most of these viruses are phages, which exhibit two primary lifestyles: virulent phages undergo exclusively lytic cycles resulting in the release of phage particles through bacterial lysis; temperate phages can either enter a lytic cycle or establish lysogeny, a dormant state in which the phage, now termed a prophage, either integrates into the bacterial genome or persists as an episome. Upon exposure to specific environmental cues prophages may reactivate, resuming their infection leading to bacterial lysis and virions release.^46^

In OMM^12^ mice, phage abundance is estimated at 10^8^ VLPs per gram of feces, and active prophages have been identified through complementary sequencing approaches.^32^ ^,^ ^47^ ^,^ ^48^ Bioinformatic predictions from the twelve bacterial genomes identified 44 putative prophages. However, proximity ligation-based sequencing (Hi-C) revealed that only 16 of these prophages exhibited 3D structural signatures indicative of induction. Subsequent sequencing of VLPs (virome) of fecal samples and *in vitro* cultures confirmed the production of viral particles for 11 prophages *in vivo* and/or *in vitro* and further highlighted two additional induced prophages.^32^ Collectively, the OMM^12^ microbiota comprises 13 induced prophages, derived from 7 bacterial strains, which were named after French castles: Versailles (*B. caerimuris* I48), Chambord, Chantilly, Moulinsart (*A. muciniphila* YL44), Saumur, Villandry (*E. clostridioformis* YL32), Castelnaud, Chenonceau, Cormatin (*F. plautii* YL31), Amboise, Maintenon (*C. innocuum* I46), Montmirail (*B. coccoides* YL58) and Fontainebleau (*A. muris* KB18).

Moreover, unmapped reads to the 12 bacterial genomes from virome sequencing failed to identify either virulent phages or eukaryotic DNA viruses, demonstrating that the OMM^12^ virome is exclusively composed of temperate phages.^32^

While DNA-damaging agents such as mitomycin C or UV radiation are well-established *in vitro* inducers of prophages, the environmental cues triggering prophage induction *in vivo* remain poorly characterized.^49^ Strikingly, some prophages exhibit context-dependent induction patterns: Fontainebleau, Moulinsart, and Amboise were spontaneously induced *in vitro* but not *in vivo*, whereas Saumur was induced *in vivo* but not *in vitro*.^32^ These observations suggest that gut-specific factors, whether inhibitory or stimulatory, regulate prophage induction, underscoring the necessity of virome sequencing to identify which temperate phages are actively released before undertaking quantitative analyzes.

### The host

Beyond microbial relationships, the OMM^12^ model has served as a powerful platform for investigating the interplay between the gut microbiota and host physiology, including enteric-associated neuronal activity, development, immune homeostasis and metabolism.^27,37,50–53^ In particular, the gut microbiota plays a pivotal role in priming, regulating, and maintaining immune system homeostasis (as reviewed by^54^). Notably, OMM^12^ mice exhibit distinct immune features compared to SPF and GF mice, including altered densities of specific immune cell populations, altered differentiation and variations of antibody levels.^29,55–58^ Accordingly, these differences are reflected at the transcriptional level of genes involved in inflammatory cell signaling.^37^ ^,^ ^57^ The OMM^12^ model was also pivotal to demonstrate a link between metabolic perturbation of epithelial cells and overgrowth of *B. caecimuris*, which cooperate in the development of metabolic injury during intestinal inflammation.^53^

The intermediate complexity of the OMM^12^ microbiota, together with the absence of key microbial signals, creates an “intermediate state” that offers a unique opportunity to investigate microbiota–immune system interactions. A compelling example is the study of immunoglobulin E (IgE).^29^ The level of this antibody is typically elevated in allergic conditions but also in GF mice. Interestingly, in OMM^12^, IgE level is significantly reduced compared to GF animals, though still higher than in SPF mice. This indicates that the minimal OMM^12^ community is sufficient to partially suppress hyper-IgE phenotype.^29^ Given the critical role of early-life colonization in shaping adult IgE levels, subsequent experiments with OMM^12^ mice identified strains highly abundant before weaning, including *A. muciniphila* YL44 and *E. faecalis* KB1. Mono- or co-colonization experiments showed that these strains suppressed hyper-IgE only when combined with *B. coccoides* YL58. Mechanistic studies further revealed that this suppression relies on acetate production by YL58 and IgA stimulation by the two other strains, demonstrating functional cooperation among OMM^12^ members.

Overall, the OMM^12^ mouse line has proven invaluable for studying multipartite interactions between microbial components and the host physiology.^50–52,56,59^ Moreover, it has been extensively used as a versatile platform to investigate the functional impact of additional bacterial strains, whether commensals or pathogens, as will be detailed in the next section.

## II - The OMM^**12**^ model as a platform for dissecting gut microbiota functionalities

### Gut colonization and microbial interactions

One major advantage of the OMM^12^ model lies in its straightforward complementation with a broad range of additional microbial strains. While only two studies have reported failed colonization, possibly due to competition with the resident community or host-mediated exclusion mechanisms,^36^ ^,^ ^60^ over 22 different bacterial species belonging to 17 families and 6 phyla have been successfully introduced to the OMM^12^ community to date. Moreover the model has been expanded to include one archaeon and five yeast strains^61^ ^,^ ^62^ (Table 1). Notably, unlike in SPF mice, antibiotic pretreatment is not required in OMM^12^ mice to disrupt the resident microbiota and alter gut homeostasis to create favorable ecological niches for introduced bacteria.^25^ This simplifies experimental workflows while minimizing confounding effects associated with antibiotic-induced dysbiosis.

**Table 1.** List of microbes that have been introduced to complement the OMM^12^ consortium.

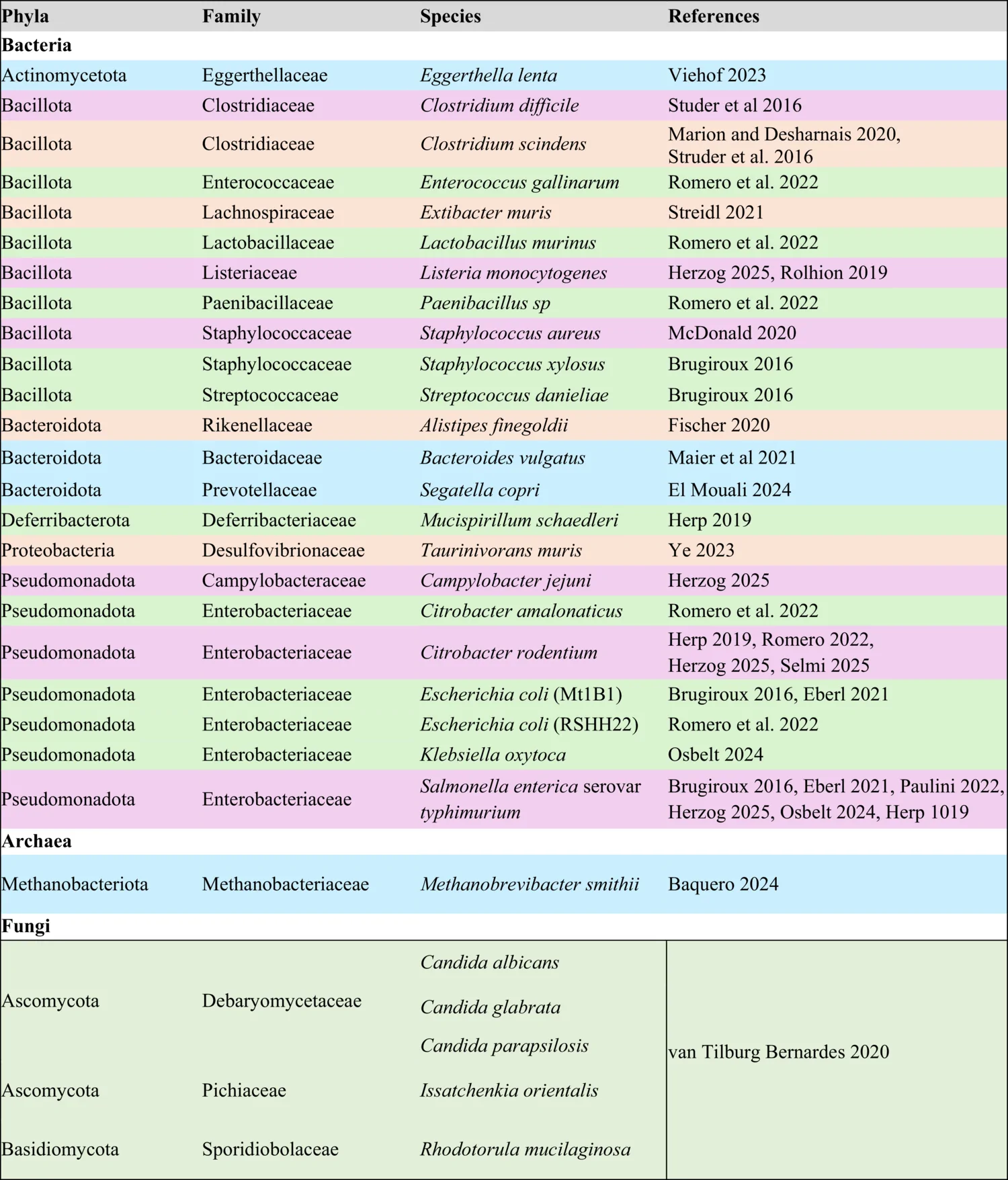

Blue, intestinal colonization; pink, host-response to pathogen; green, microbiota-derived protective mechanisms against pathogen or inflammation; orange, additional metabolic function.

#### Identification of bacterial factors sustaining gut colonization

As a reductionist model, the colonization of OMM^12^ mice with additional strains provides a powerful framework for investigating the mechanisms underlying enteric colonization. A striking example is the case of *Segatella copri*, which fails to colonize GF mice maintained on a standard chow diet but successfully establishes in OMM^12^ mice under identical dietary conditions.^33^ ^,^ ^63^ This observation not only underscores the role of the ecological context provided by the resident OMM^12^ community but also demonstrates that a defined yet functionally active microbial background can overcome dietary barriers that would otherwise prevent colonization in GF settings. Leveraging this model, a non-coding RNA identified in human microbiomes studies (*SrcF*) was also transcribed in OMM^12^ mice.^33^ Co-colonization experiments further revealed that wild type *S. copri* outcompeted its Δ*SrcF* mutant by 1,000-fold at day 14 post-colonization, demonstrating that this non-coding RNA is essential for gut colonization. Studies investigating colonization factors are particularly powerful in SynCom models because the defined resident community offers a dual advantage of its permissiveness to a broad range of new colonizers and its ecological relevance as it mimics realistic niche competition and community dynamics.

#### Dissecting mechanisms of colonization resistance

A fundamental function of the gut microbiota is to protect against the invasion and expansion of undesirable bacteria, including pathogens. This process, known as colonization resistance (CR), operates either by direct mechanisms including killing of pathogens and competition for niche and resources, or by indirect mechanisms mediated by the host immune responses reviewed in.^64^

Early studies in ASF mice revealed that expanding the consortium with diverse strains was insufficient to prevent *Salmonella spp.* infection, in contrast to observations in conventional mice.^65^ This highlighted the need to identify key missing functions in gnotobiotic communities and was a critical consideration in the early development of the OMM^12^ model. A proof-of-concept study demonstrated that OMM^12^ mice, when complemented with three murine-derived aerobic strains (including *Escherichia coli* Mt1B1), achieved CR against *Salmonella enterica* serovar Typhimurium (S. Tm).^18^ Subsequent studies showed that *E. coli* Mt1B1 alone, as well as a *Klebsiella oxytoca* strain, was sufficient to confer this protection.^31^ ^,^ ^66^

The OMM^12^ model has enabled researchers to move beyond descriptive studies of the protective phenotypes and dissect underlying mechanisms using advanced experimental and -omics technologies. First, metabolomic profiling of the OMM^12^ consortium (described in **Section I**) has provided a foundation for testing the roles of specific metabolic pathways absent in the model. For instance, production of secondary bile acids *via* 7α-dehydroxylation of primary bile acids is restricted to a limited set of bacteria, absent from the OMM^12^ consortium. In conventional mice, secondary bile acids inhibit *Clostridioides difficile* growth and spore germination, possibly explaining the lack of CR against *C. difficile* in OMM^12^ mice.^25^ ^,^ ^67^ ^,^ ^68^ Consistent with this hypothesis, complementation with *Clostridium scindens* resulted in transient reduction of early *C. difficile* colonization. However, this intervention failed to prevent *C. difficile* infection, as no significant differences were observed compared to controls lacking *C. scindens* at 72 hours post-infection.^25^ While the long-term sufficiency of bile acid-induced gene expression in controlling *C. difficile* remains unclear, these findings demonstrate the utility of the OMM^12^ model for enhancing metabolic potential and linking nutrient competition to pathogen resistance mechanisms. Second, combined transcriptomic analysis revealed that in OMM^12^ mice, both *E. coli* Mt1B1 and S. Tm upregulate dulcitol (or galactitol, a sugar produced through the metabolism of galactose) utilization genes, a response not observed in ASF mice, where Mt1B1 fails to confer CR to S. Tm.^31^ The authors demonstrated that competition for this carbohydrate, which is not catabolized by the OMM^12^ members, drives the outgrowth of Mt1B1 over S. Tm. Interestingly, dulcitol catabolism also contributes to *K. oxytoca* ability to outcompete S. Tm *in vivo*.^66^

Additionally, a key advantage of the OMM^12^ model is the ability to selectively deplete its bacterial consortium. Notably, *E. coli* Mt1B1 failed to prevent S. Tm invasion in mice lacking *E. clostridioformis* YL32 and *B. coccoides* YL58 (OMM^11^).^31^ This highlighted the necessity of additional commensal interactions for effective CR, further validating the relevance of the OMM^12^ model in studying microbial cooperation.

Finally, competitive interactions beyond CR can be dissected in OMM^12^ mice, highlighting particularly the influence of the microbial context on the nature of bacterial competition For example, while the production of the toxin tilimycin is critical for *K. oxytoca* to compete with S. Tm in GF mice, these effects are less pronounced in OMM^12^ mice.^66^ The authors suggested that higher levels of carbohydrates in GF mice compared to OMM^12^ mice are likely minimizing nutrient competition. This highlights the enhanced ecological relevance of using stable synthetic communities to study gut bacterial interactions. Competitive interactions associated with various mechanisms and effects on gut colonization have been uncovered within OMM^12^ mice. For instance, prior colonization with *Mucispirullum schaedleri* (*Deferribacterota*) reduced colitis severity in S. Tm-infected OMM^12^ mice. Transcriptomics analyzes highlighted nitrate competition as a key mechanism in downregulating S. Tm virulence. This direct commensal-pathogen interaction for reducing virulence was also validated in the ASF model.^69^ The OMM^12^ model was also instrumental in supporting the exacerbating role of the commensal *Prevotella copri* in intestinal infection by *Listeria monocytogenes*. By secreting the Lmo2776 bacteriocin, *L. monocytogenes* reduces *P. copri* abundance to avoid excessive inflammation that is reducing *L. monocytogens* colonization.^70^ This exemplifies a microbiota-dependant modulation of gut pathogen colonization, with key intestinal species critically influencing infection outcomes.

Overall, the OMM^12^ model is emerging as a referenced framework to investigate mechanisms of gut colonization. It has been particularly useful to identify critical species and functions for CR and to highlight the importance of microbial context in protective outcomes. However, CR studies have primarily focused on S. Tm. Expanding these investigations to other pathogens, such as *Campylobacter jejuni*, which infects OMM^12^ but poorly colonize SPF mice, could uncover additional CR mechanisms.^71^ Dulcitol consumption identified as a driver of the competion between S. Tm and either *E. coli* Mt1b1 or *K. oxytoca,* calls for a broader evaluation of this pathway towards other pathogens. Whether metabolic competition is a dominant mechanism or is this mechanism easily assessed in models displaying moderate complexity in metabolic networks remains to be clarify. Nevertheless, studies with conventional mice have also identified dulcitol competition between *E. coli* spp and *Klebsiella* spp, and another study highlighted galactose as a key nutrient mediating CR against S. Tm.^72^ ^,^ ^73^ Finally, the role of the host immune system in CR warrants deeper investigations to understand how commensal strains prevent pathogen colonization.

### Host responses to bacterial colonization

#### Dissecting strain-specific immune modulation

The reproducibility and defined composition of the OMM^12^ model have established it as a reproducible biological system for investigating how individual strains influence host immune responses. The cellulolytic strain *Alistipes finegoldii* increased the IL-22 production and antibacterial peptide Reg3γ expression in the colon, which are critical factors for maintaining bacterial compartmentalization within the gastrointestinal tract.^42^ Thus, enrichment of the OMM^12^ gut metabolic capacity enables dissecting specific effects of metabolic functions or by-products on the host immune system.

Moreover, the homogenous genetic background of the model allows direct comparisons of host responses to diverse pathogens. Beyond S. Tm-related infection dynamics studies, the behavior of other pathogens such as *C. jejuni*, *L. monocytogenes* and *Citrobacter rodentium* has been evaluated.^71^ ^,^ ^74^ While all three pathogens triggered inflammation, only *C. jejuni* elicited an adaptative response in the cecum, highlighting pathogen-specific signatures leading the authors to emphasize the potential of the OMM^12^ model for standardizing research in the gut infection field.^71^

While host immune barriers play a critical role in CR, they have been investigated in only a limited number of studies, with one notable exception: CR against *C. rodentium*, a murine pathogen that mimics Enteropathogenic (EPEC) and Enterohemorrhagic *E. coli* (EHEC). In SPF mice, *C. rodentium* is cleared while in GF mice it persists. In contrast, in OMM^12^ mice, *C. rodentium* exhibits a unique intermediate phenotype, characterized by a rapid colonization followed by a steady decline to a plateau, with subsequent long-term persistence.^35^ ^,^ ^57^ This distinctive pattern phenotype makes the model a valuable framework for deciphering the relative contributions of the resident microbiota and the host immune responses in shaping infection outcomes.

#### Microbiota-mediated modulation of pathogen virulence and host response

To investigate how the gut microbiota indirectly influences microbe pathogenicity by shaping host immune responses to infections, the OMM^12^ model can be directly compared to other models. For instance, in IL10^-/-^ mice, both GF and ASF animals developed colitis upon infection with murine norovirus (MNV), whereas OMM^12^ mice remain protected.^60^ However, whether this protection correlates with the higher pro-inflammatory cytokine levels previously reported in OMM^12^ mice compared to ASF mice remains to be elucidated. The OMM^12^ model also exhibits distinct responses to *Lymphocytic choriomeningitis* virus (LCMV) infection, setting it apart from both GF and SPF mice. OMM^12^ mice display greater body weight loss and enhanced viral clearance that were associated with increased proliferation and functionality of T helper type 1 cells.^55^ Moreover, a recent untargeted metabolomic analysis of *C. rodentium*-infected OMM^12^ mice identified a specific ornithine lipid produced by *A. muciniphila* YL44 as a novel immunoregulatory molecule that potentially regulates intestinal inflammation.^35^ This suggests that some members of the OMM^12^ consortium enhance host immune responses by mechanisms that are just starting to be uncovered.

Expansion of the OMM^12^ model also enables further dissection of the mechanisms underlying CR against pathogens. Pre-colonization with *Citrobacter amalonaticus* prior to *C. rodentium* infection was sufficient to achieve pathogen clearance with dynamics comparable to those observed in SPF mice.^57^ This clearance is proposed to be mediated by enhanced angiogenesis and increased neutrophil extravasation into the lamina propria and lumen of the colon. This shows that immunity of the OMM^12^ can be precisely modulated through defined microbial complementation, thereby dissecting microbiota-host interactions.

Moreover, metabolites produced by the OMM^12^ consortium were sufficient to restore the proliferation of an auxotrophic S. Tm strain—virulent yet non-inflammatory - while its colonization in GF mice remained transient.^75^ This experiment enabled decoupling bacterial pathogenicity from the inflammatory response it elicits. The authors further showed that protection against wild-type S. Tm arises from a synergy between IgA-mediated immune pressure (dependent on virulence factors) and bacterial niche exclusion.^75^ Thus, the OMM^12^ model highlighted how immune mechanisms and bacterial competition jointly shape pathogen control.

Importantly, the utility of the OMM^12^ model extends beyond gut-restricted infections. For instance, McDonald et al.^76^ showed that 100% of OMM^12^ mice survived intravenous *Staphylococcus aureus* infection, while this rate dropped to 80% of SPF mice and 20% of GF mice.^76^ This study further showed that lactate produced by the microbiota enhances ROS-mediated killing of *S. aureus* by Kupffer cells. This study, so far unique, demonstrates that OMM^12^ mice can uncover microbial components involved in microbiota-dependent immune responses for pathogen clearance outside the gut.

Future research employing the OMM^12^ model, including co-infections, host cytokine profiling, and immune cell population monitoring will deepen our understanding of how defined microbial communities modulate both innate and adaptive immunity. Beyond host-bacterial interactions, the OMM^12^ model has also facilitated investigations into the viral component of the microbiota, enabling further characterization of phage-bacteria-host dynamics.

### Viral dynamics in the OMM^**12**^ model: from prophages to therapeutic phages

#### *Investigating prophage induction in OMM* ^*12*^ *mice*

The defined bacterial composition of OMM^12^ mice allows investigations on the dynamics of prophages harbored by resident strains, as well as introduced microbes.^61^ This model offers a unique opportunity to explore diverse signals affecting prophage dynamics. Potential triggers for gut prophage induction comprise external factors such as diet or drugs including antibiotics, as well as signals originating from the host or the microbial community.^49^ While *in vitro* studies have established that certain antibiotics induce the SOS response, which in turn activates prophages,^77–79^ *in vivo* evidence remains scarce, with only a few studies demonstrating prophage induction in animals or humans.^80^ ^,^ ^81^ In OMM^12^ mice, Münch et al.^82^ showed that the prophage Versailles, which is carried by *B. caecimuris* strain I48, is induced following pulsed treatment with vancomycin, ciprofloxacin or tetracycline, by performing reads mapping from shotgun sequencing on the OMM^12^ genomes.^82^ Additionally, Ye et al.^43^ reported an increased transcriptional activity of prophage Saumur (*E. clostridioformis* YL32) in OMM^12^ mice colonized with H_2_S-producing *T. muris*, including prophage-encoded genes involved in sulfur metabolism.^43^ However, *in vitro* assays failed to confirm a direct role of H_2_S in triggering Saumur induction, suggesting that the actual triggering molecule may be a distinct metabolic by-product. Future investigations leveraging the controlled ecosystem of OMM^12^ mice will be critical to identify both triggering and repressing factors and the molecular mechanisms underlying these processes.

#### *Therapeutic and ecological perspectives of virulent phages in OMM* ^*12*^ *mice*

Over the past decade, phage therapy, the use of virulent phages to target antibiotic-resistant pathogens, has gained increasing support with numerous successful compassionate treatments reported.^83^ Beyond targeting pathogens during intestinal infections, virulent phages have also been proposed as a precision tool to target undesirable members of the gut microbiota. A prophylactic phage treatment targeting S. Tm showed its tolerability and efficacy to reduce S. Tm loads in OMM^12^ mice.^84^ A precision treatment with phage cocktails targeting either *E. coli* Mt1B1 or *E. faecalis* KB1 in OMM^13^ (12 strains + Mt1B1) mice following S. Tm infection resulted in a decreased CR.^85^

However, virulent phage application does not necessarily result in bacterial clearance. For example, long-term stable coexistence between *E. coli* Mt1B1 and its targeting phages was observed in OMM^12^ mice, without the emergence of resistant bacteria.^86^ Instead, this coexistence was maintained through source-sink dynamics, with bacteria residing in phage-inaccessible mucosal sites.^86^

More broadly, phage application in the OMM^12^ mice provides a tool for investigating microbial interactions with therapeutic and ecological implications. Whether for deciphering molecular mechanisms involved in a specific phage-pair system, or for evaluating the broader impact of metabolic shifts or host responses, such as inflammation, the reductionist approach offered by the OMM^12^ model overcome limitations of SPF mice or humans studies, where the complexity and uncharacterized nature of resident phage community often hinder mechanistic insights.^87^ ^,^ ^88^

## Conclusions and limitations

Over the past decade, the OMM^12^ model has emerged as a cornerstone in gut microbiota research, effectively bridging the gap between oversimplified monoxenic systems and complex conventional models. The versatility of this model has been demonstrated across diverse research questions, highlighting its reproducibility (consistent colonization dynamics), modularity (ability to add/remove strains) and adaptability (to different host backgrounds).^28^ ^,^ ^29^ ^,^ ^31^ ^,^ ^41^ ^,^ ^53^ ^,^ ^60^ Its reductionist yet functionally competent design has enabled precise dissection of strain-specific contributions to host-microbiota interactions and controlled manipulation of microbial communities to study ecological and metabolic networks (Figure 3).

Like all experimental models, the OMM^12^ has inherent limitations that must contextualize findings. Sterile housing requirements—including air-flow isolators, biosafety cabinets, strict protocols and regular contamination monitoring—make the model costly, labor-intensive and requires trained users.^89^ ^,^ ^90^ Biological constraints include absence of *Enterobacteriaceae*, limiting pathogen competition (e.g., *S. Tm*) as well asmetabolic gaps, such as missing 7α-dehydroxylation of primary bile acids.^25^ Colonization challenges can constitute additional constraints, e.g., SFB requiring pre-colonization and resident community outcompetition of introduced strains.^60^ Finally, diet is a critical variable to consider for standardizing bacterial composition, as evidenced by studies of dietary shifts and/or using diets other than standard chow.^30^ ^,^ ^40^

These biological limitations have spurred the development of modular OMM^12^-based platforms. For example, the OMM^19.1^ model adds strains to enhance SCFA production and mucus turnover, expanding immune/metabolic functionality.^22^ ^,^ ^91^ Standardized strain collections (e.g., miBC) now offer “ready-to-use” microbiota mixes (including OMM^12^/OMM^19.1^), enabling cross-study comparability.^23^ ^,^ ^92^ Additionally, new SynComs have been developed, such as the GM15 mouse line, which comprises 15 murine strains with partial overlap with the OMM^12^ consortium, which exhibits distinct immune phenotypes and growth dynamics,^93^ whereas the PedsCom, a nine-strain consortium mimicking the pre-weaning murine microbiome, has enabled studies of early-life microbiota.^59^ ^,^ ^94^ Dissecting key differences between SynComs will improve our understanding of host-microbe co-adaptation and microbial community assembly.

Finally, SynComs models provide an ideal platform to integrate *in vivo* multi-omics approaches, develop quantitative imaging and assess controlled perturbations (e.g., diet, phages) to translate these novel insights into clinical applications, ultimately advancing our ability to treat and prevent microbiota-associated diseases. These models can also be used to test strategies to mitigate collateral depletion using antibiotics^95^ and assess alternatives to antibiotics including microbiota-based therapies (bacterial-derived metabolites), precision bacterial targeting by phage therapy or bacteriocin-producing probiotics.^96–98^ Looking forward, it is anticipated that next-generation SynComs will more extensively integrate understudied microbiota members, including viruses, archaea and fungi, to reveal their specific contributions in health and disease.^61^ ^,^ ^62^
